# Applying a GM (1, 1)-BPNN to predict pavement Rutting Depth Index in hot and humid region: A case study in Guangdong, China

**DOI:** 10.1371/journal.pone.0326340

**Published:** 2025-07-03

**Authors:** Guodong Zeng, Yixi Hu, Hao Li, Yonghong Yang, Xuancang Wang

**Affiliations:** 1 Foshan Transportation Science and Technology Co., Ltd., Foshan, China; 2 School of Civil Engineering and Transportation, South China University of Technology, Guangzhou, China; 3 School of Highway, Chang’an University, Xi’an, China; University of Wisconsin-Milwaukee, UNITED STATES OF AMERICA

## Abstract

Pavement performance prediction plays a crucial role in formulating scientific pavement maintenance plans. However, current research on how the rutting depth index (RDI) in hot and humid regions is affected by multiple influencing factors and the development of accurate prediction indicators remains insufficient. To establish a scientific basis for maintenance, the research team collected maintenance, traffic, pavement surface and internal temperature, climate, and road condition data from 2015 to 2021 for a freeway section located in Foshan, China, a typical hot and humid region. Then, a combined predictor, GM(1,1)-BPNN, was proposed to conduct accurate RDI prediction for the pavement. Furthermore, the SHapley Additive exPlanation (SHAP) method was employed to analyze the impact of each influencing factor on RDI in greater detail. The results indicated that 1) The proposed combined model has a higher prediction performance. Validated by validation set, the MAE, MSE, RMSE as well as R^2^ were 0.068, 0.004, 0.068, 0.79, respectively, surpassing the baseline models PPI and GM (1, 1); 2) The SHAP analysis shows that maintenance fund, middle layer maximum temperature, integrated radiation, and pavement surface maximum temperature have a more significant impact on RDI. The conclusions of the paper provide a theoretical basis for road administrations to formulate scientific maintenance plans and contribute to understanding the impact of climatic and traffic environments on RDI.

## Introduction

With the development of the economy and society, infrastructure continues to improve, and the highway network is becoming increasingly dense. According to Chinese statistic yearbook, by the end of 2023, the total mileage of national roads had reached 5.44 million kilometers, with the total mileage of expressways being 183 thousand kilometer [1]. However, the continuously increasing road mileage also puts tremendous pressure on road maintenance. At present, newly constructed road surfaces are mainly asphalt pavement, which, compared to cement concrete surfaces, has the advantages of a smooth surface without seams, less vibration during driving, lower noise, faster opening to traffic, and easier maintenance [2,3]. However, with the increase in the number of years in service, if not properly maintained, asphalt pavements are prone to cracks, potholes, and other diseases, which can affect driving comfort and safety. According to relevant departments, China needs to carry out medium and major repairs on about 200,000 kilometers of ordinary roads and more than 10,000 kilometers of expressways every year [4], consuming a huge amount of manpower, material resources, and financial resources.

Asphalt pavement performance prediction refers to the process of using historical asphalt pavement performance indicators and factors affecting the performance of asphalt pavements, selecting mathematical models and machine learning models for modeling and prediction, which is of great significance for scientifically formulating asphalt pavement maintenance plans [5–7].

In 2021, commissioned by Foshan Traffic Technology Co., Ltd., the research team carried out pavement performance prediction work on pavements under the company’s jurisdiction. Due to data confident policy, this paper uses Freeway A to denote the research freeway. This freeway located in Foshan City, Guangdong province, southern part of China, which is a hot and humid area with high temperatures and high humidity. Previous researches have shown that asphalt pavements in these regions are prone to aging, rutting, and cracking [8].

After a detailed survey of road conditions, it was found that the rutting disease along Freeway A is severe, which would do harm to vehicles safe passage [9]. To formulate a scientific asphalt pavement maintenance plan and explore the impact of various factors on the rutting disease of asphalt pavements in hot and humid regions, this paper collects data related to the Rutting Depth Index (RDI) and factors affecting RDI, which is a commonly used pavement technical evaluation index in China [10]. Additionally, a combined predictor consists of grey forecast model and machine learning techniques was proposed to forecast RDI.

The main contributions of this paper are as follow:

1. This paper proposes a RDI predictor combining GM (1,1) and BP neural network, which comprehend the goodness of grey forecast model and machine learning methods.
2. By field survey, the research team obtained 15 types of environment data, and applied BP neural network and SHAP technique to provide insight on how these environment indicators affect RDI.

The outcome of this paper can provide a scientific basis for highway maintenance agencies to make maintenance decisions and help learn about how environmental factors affect pavement RDI.

## Related works

Accurate prediction of road performance is essential for establishing a scientific basis for pavement maintenance strategies [11–13]. Effective predictive models enable road administration to better identify maintenance needs, allocate funds reasonably, and develop suitable maintenance plans [14–16]. At present, commonly used pavement performance prediction models domestically and internationally include mechanistic models, empirical models, mechanistic-empirical models, machine learning models, and probabilistic models [17].

In prior research, scholars have investigated various models. Zhao et al. [18] applied the same-dimension gray recurrence dynamic model to predict pavement PCI, RQI, and SRI, demonstrating acceptable accuracy across all three indicators. Ziari et al. [19] employed Artificial Neural Networks (ANN) and Group Method of Data Handling (GMDH) to predict pavement performance indicators over three forecasting periods. They used nine input variables classified into traffic conditions, environmental changes, and pavement structure and found that ANN models performed well in both short- and long-term predictions, while GMDH models showed less satisfactory accuracy. Zhang et al. [20] used the K-Nearest Neighbors (KNN) algorithm to predict the performance of typical asphalt pavements on national and provincial roads in Guangdong Province, achieving high prediction accuracy. Deng and Shi [21] proposed a neural network model optimized using the Particle Swarm Optimization (PSO) algorithm, finding that their PSO-NN model achieved a balance in terms of accuracy, reproducibility, and robustness. Similarly, Xiao et al. [22] developed a Back Propagation Neural Network (BPNN) optimized with PSO, considering variables such as surface layer thickness, traffic load, and climatic conditions to predict six asphalt pavement performance parameters. Li et al. [23] proposed an innovative Particle Swarm Optimization (PSO) algorithm-enhanced two-stage TrAdaBoost.R2 transfer learning algorithm for predicting pavement performance, and found that the proposed PSO-Two-stage TrAdaBoost.R2 model has the highest accuracy compared to the AdaBoost.R2 model and traditional regression decision tree models. Their model demonstrated faster convergence and improved prediction and generalization performance. Wang et al. [24] proposed a grey prediction model with variable weight evaluation for asphalt pavement performance, forecasting the performance of typical pavements in humid and hot regions and providing relevant maintenance recommendations. Zhao et al. [25] introduced a Grey Relational Analysis-Support Vector Machine (GRA-SVM) model to predict asphalt pavement performance using the RDI values of a highway, demonstrating good accuracy and operability. The mentioned studies have proposed various models to predict different pavement performance indicators and achieved certain results. However, most of these studies either rely solely on single mathematical models to simulate the deterioration patterns of pavement performance or limit themselves to nonlinear models for analyzing the interactions of multiple factors, failing to effectively bridge the two approaches to combine their advantages.

The performance of asphalt pavements is influenced by numerous factors. Based on the above models, past research has comprehensively analyzed these factors, which can be broadly categorized into pavement distress data, traffic volume data, historical maintenance records, and climatic and environmental data. For instance, Zhao et al. [25] investigated the effects of equivalent axle loads, maintenance funds, maximum annual temperature, rainfall, and sunlight on RDI. Xiao et al. [22] examined the impacts of traffic load, low-temperature days, and high-temperature days on asphalt pavement performance. Taheri and Sobanjo [26] explored the relationships between pavement structural performance and five categories of factors: construction parameters, construction quality, climate, traffic, and service conditions. Marcelino et al. [27] analyzed the effects of pavement structure, climate, and traffic conditions on IRI. The foregoing researches investigates the relationship between various features and pavement serviceability and finds that these features all have significant impact on pavement serviceability. For example, Deng and Shi [28] found that factors such as AADTT, bulk specific gravity of aggregate, and surface evaporation significantly influence rutting performance, while maximum and minimum daily temperatures have a more pronounced effect on IRI. Moreover, several studies examined the relationship between pavement distress severity and performance metrics. For example, Naseri et al. [29] explored the impact of alligator cracking, block cracking, and bleeding on IRI. Gong et al. [30] studied the influence of fatigue cracking, raveling, potholes, polishing, as well as structural, climatic, and traffic factors on IRI. Although these studies explored various factors influencing pavement performance, they did not specifically address the environmental conditions of different pavement layers. Some of the mentioned papers are summarized in Table 1.

**Table 1:** Summary of related works.

Author	Predicted Indicator	Model	Features
Xiao et al. [22]	RDI, RQI, PCI, SRI, PQI, PSSI	PSO-BPNN	Road performance, Traffic load, Temperature, Road age.
Zhang et al. [20]	PQI	KNN	N/A
Zhao et al. [18]	PCI, RQI, RDI, SRI	GM(1,1)	N/A
Ziari et al.[19]	IRI	ANN, GMDH	ESAL, PT,AADT, AADTT, AADI.
Gong et al. [30]	IRI	Random Forest	Pavement structure, Pavement performance, Traffic condition, Climate condition.
Fang et al. [31]	RDI	Wavelet-time series prediction model	N/A
Sun et al.[32]	PCI, RQI, SRI, RDI	LSTM	Road age, Climate, Pavement structure, Traffic condition
Deng and Shi [21]	RD	PSO-NNS	Material properties, Structural parameters, Traffic conditions, Environment conditions.
Wang et al. [24]	PCI, RQI, RDI, SRI, PBI	GM(1,1)	N/A
Fakhri et al. [33]	IRI	ANN	Longitudinal cracks length, Transversal cracks length, Pattern cracking percentage in 10m section, Ratio of length of all cracks to section area in 10m section, etc.
Zhao et al. [25]	RDI	GM (1, 1) – SVM	Road age, Equivalent axes load, Maintainance fund, Temperature, Raindrop, Daylight.

Overall, existing pavement performance prediction methods have limitations in long-term forecasting and adaptability to environmental factors. Most traditional methods rely on static or simplified dynamic assumptions, making it difficult to accurately capture the nonlinear deterioration trends of pavement performance over time. The degradation of pavement performance is influenced by multiple factors, including traffic loads, climatic conditions, material properties, as well as other factors such as investments from road maintenance management units. However, existing empirical models (e.g., the PPI model) often account for only one or a few factors (e.g., pavement age and regional influences) while neglecting the effects of other dynamic variables. Based on the reviewed literature, this study proposes a combined GM(1,1) – BPNN predictor to forecast the RDI index of highways in humid and hot regions. Additionally, by installing temperature sensors in roadways, this study aims to further investigate the impact of roadway temperature on RDI in greater depth.

## Data preparation

### Research object

#### Road characteristics.

**Fig 1:**
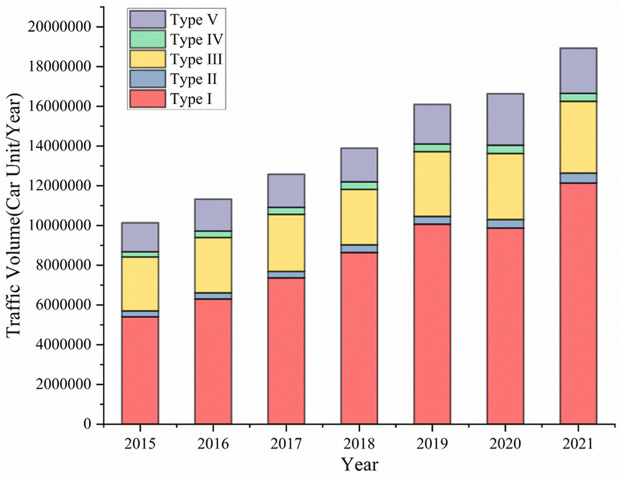
Traffic volume of Freeway A from 2015 to 2021.

**Fig 2:**
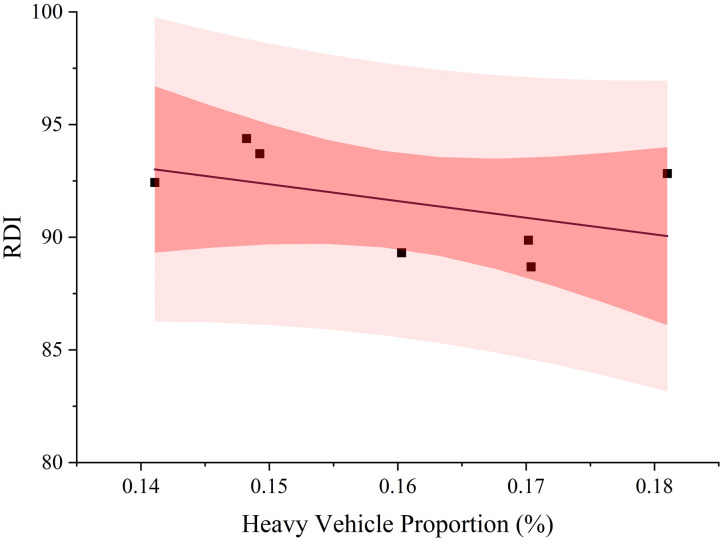
Heavy vehicle proportion from 2015 to 2021.

Initially, the basic information and pavement structure and of Freeway A is shown in Table 2 and 3, respectively. The Freeway A, located in Foshan City, Guangdong Province, China, was opened to traffic in 2011. Its design speed is 100 km/h and has a total length of approximately 135 kilometers, with six lanes in two directions. Fig 1 shows the traffic volume of different type of vehicle. Type I refers to sedans and small passenger vehicles with 7 seats or less, small trucks with a weight of 2 tons or less; Type II refers to passenger vehicles with 8–19 seats, and trucks weighing between 2 and 5 tons; Type III refers to passenger vehicles with 20–39 seats, trucks with a weight of 5–10 tons, and 20-foot container vehicles; Type IV refers to passenger vehicles with 40 seats or more, trucks with a weight of 10–15 tons, and 40-foot container vehicles; Type V refers to trucks exceeding 15 tons. Overall, the annual traffic volume steadily increased from 2015 to 2021, with an average of approximately 14.23 million passenger car units per year. The chart shows an upward trend in traffic volume on the expressway, with growth observed across all vehicle types, particularly for Type 1, which exhibited the most significant increase. The rising traffic volume poses a major challenge to road pavement service levels. Further, heavy vehicles are deemed to have more significant impact on the deterioration of pavement serviceability, as a result, Fig 2 displays the relationship between the proportion of heavy vehicles and RDI. Furthermore, the increasing proportion of heavy vehicles has led to a continuous decline in the RDI (Rutting Depth Index) and exacerbated rutting damage on the road. Based on traffic surveys, this road is classified as a heavily trafficked highway. After nearly a decade of service, significant pavement distress has become evident in various sections, which will be discussed in detail in the subsequent pavement condition survey section.

**Table 2:** Basic information of the research freeway.

Indicator	Value	Indicator	Value
In-service year	2011.12	Design Speed	100km/h
Lane number	3/direction	In-service Mile	134.924
Average traffic volume	14.23 million pcu/year	Average Truck Proportion	15.87%

**Table 3:** Asphalt pavement structure of Freeway A.

Layer	Depth & Materials	Layer	Depth & Materials
Up layer	4 cm SMA-13C	B ase	36 cm Cement stabilized macadam
Middle layer	6 cm AC-20C	Sub-base	20 cm Cement stabilized macadam
Sub layer	8 cm AC-25C	Function layer	15 cm Graded macadam

#### Pavement conditions.

Regarding pavement structure and materials, the road adopts a semi-rigid base – asphalt pavement structure. The materials and thicknesses of the respective layers are presented in Table 3. The sub-base consists of 20 cm of cement-stabilized macadam, and the base layer is composed of 36 cm of cement-stabilized macadam. The asphalt layers are divided into three layers with thicknesses of 4 cm, 6 cm, and 8 cm, respectively. The up layer uses SMA-13C, the middle layer uses AC-20C, while the sub layer uses AC-25C. Additionally, the functional layer comprises 15 cm of graded macadam.

**Fig 3:**
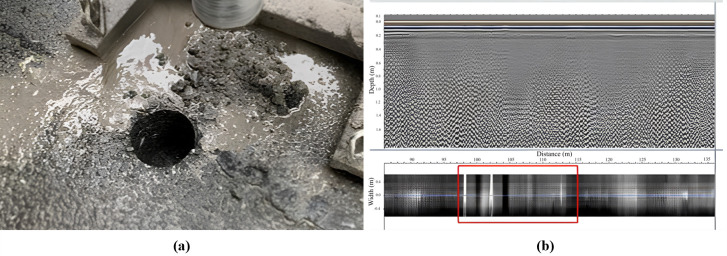
Pavement serviceability survey of Freeway A: (a) Drilling at the location of lateral cracking; (b) Indicating that the pavement suffers severe rutting.

To learn about the pavement condition of the research freeway, core drilling sampling and ground-penetrating radar were used to analyze the typical diseases of the section, and some core sample analysis diagrams are shown in Fig 3, in which, Fig 3a illustrates the drilling location and process, and Fig 3b shows that the pavement has undergone rutting, which may be due to insufficient construction quality control and insufficient initial compaction. Recycled asphalt tests were conducted on the core samples to test the three major indicators of asphalt, with penetration, softening point, and ductility being 41.0, 25.0, and 63.0, respectively, indicating that the surface layer asphalt has aged.

### Data collection and overview

**Fig 4:**
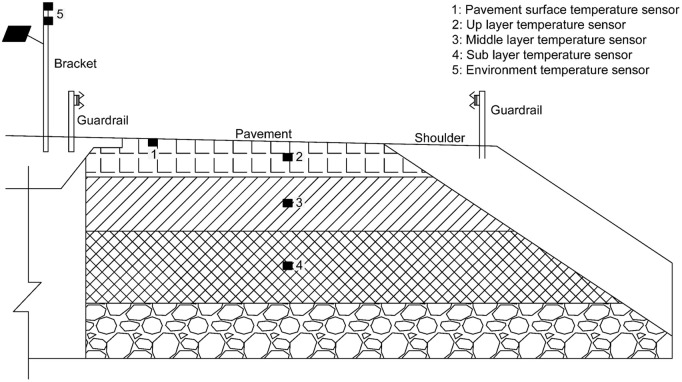
Temperature sensors’ location from the view of cross-section.

**Fig 5:**
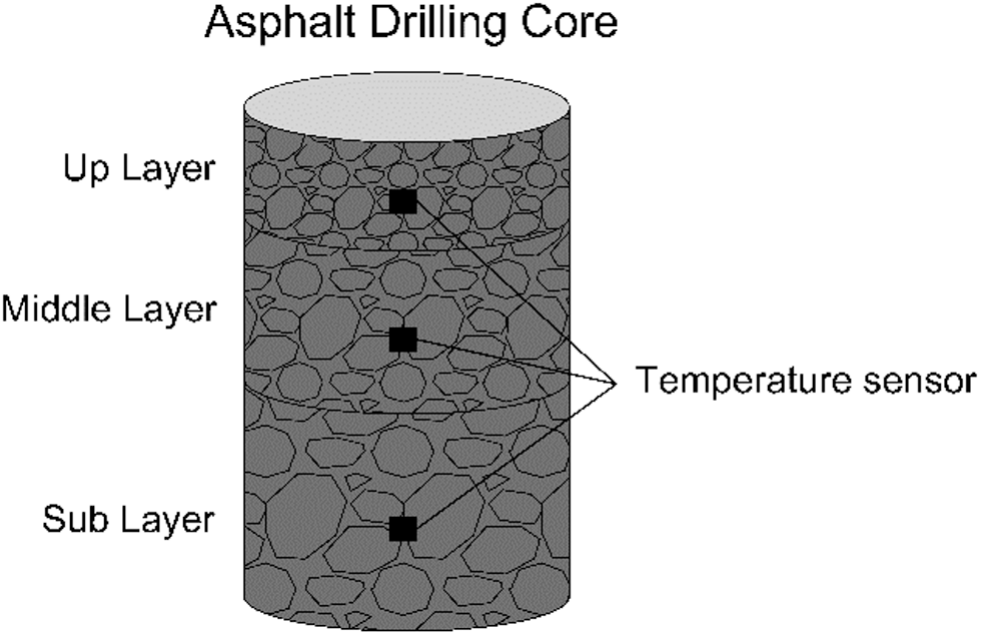
Temperature sensors located on the drilling core.

Previous research indicate that the subgrade and asphalt pavement design significantly affect pavement serviceability [34–37]. For the collection of environmental data on roads, which includes ambient temperature, pavement layer temperatures, and radiation dosage, the research team has installed several sensors along the road surface, as shown in the Figs 4 and 5. In Fig 4, specifically, a road surface temperature sensor is placed at Point 1. At Point 2, 3 and 4, we firstly drilled a core from the research freeway and place the temperature sensors in the up layer, middle layer, as well as sub layer, whose detailed image is shown in Fig 5. Consequently, backfilling the drilled core. Additionally, an environment temperature sensor was set at the roadside to collect the environment temperature. By collecting data from the mentioned sensors, the research team obtained environmental temperature, pavement surface temperature, and asphalt layer temperature data at the sampling locations.

During the data collection process, we made every effort to ensure the completeness and accuracy of the data. The research team obtained multi-source data, including road condition surveys, traffic data, and climate data, from the Foshan Municipal Transportation Affairs Center. Detailed data cleaning and screening were conducted. Additionally, box plots were generated to identify potential outliers. Following data organization and preliminary analysis, no missing data or outliers were found in the dataset used in this study.

Meanwhile, the research team obtained the pavement survey data, traffic data as well as climate data from Foshan highway administration. After data cleaning, statistical analysis, and filtering, datasets were prepared for developing the pavement prediction model. The overview of the data is shown as Table 4. Based on prior research and insights from pavement management authorities, this study selected 15 key indicators, all of which influence pavement RDI [22,25,30,33]. GP-RDI refers to the RDI value predicted using the GM(1,1) model, which will be discussed in greater detail in subsequent chapters. Traffic data primarily includes the equivalent cumulative axle load repetitions, a commonly used metric for analyzing pavement loading. Environmental factors mainly consist of annual precipitation and integrated solar radiation, both of which are potential indicators influencing pavement RDI. Temperature metrics encompass the temperatures at different pavement layers, including the surface, upper layer, middle layer, and lower layer, with their maximum and minimum temperatures recorded. These factors also empirically affect RDI. Additionally, this study considers the maintenance fund invested by road agencies as another influencing factor on RDI.

**Table 4:** Variables overview and description.

Abbreviation	Description	Min	Max	Mean	St.D
**Independent Variable**					
RDIs	Rutting Depth Index	88.690	94.380	91.603	2.107
**Explanatory Variables**					
GP-RDI	Rutting Depth Index predicted by GM(1,1)	89.320	94.290	91.764	1.903
AEAL	Accumulative equivalent axle load (Times)	1718	2926.5	2293.716	418.887
MFI	Maintenance fund input (×10^4^ CNY)	46.13	1812.1	754.991	611.547
AAR	Average annual rainfall (ml)	1392.5	2306.3	1894.300	263.846
Env_MaxT	Max environment temperature (°C)	35	38	36.714	0.881
Env_MinT	Minimum environment temperature (°C)	0	6	3.714	1.829
Pav_S_MaxT	Pavement surface Max Temperature (°C)	42	51.5	45.643	3.008
Pav_S_MinT	Pavement surface Min Temperature (°C)	4	7	5.571	0.904
UL_MaxT	Up layer Max temperature (°C)	34	36.5	35.214	0.920
UL_MinT	Up layer Min temperature (°C)	5	11	8	1.852
ML_MaxT	Middle layer Max temperature (°C)	36	41	38.571	1.591
ML_MinT	Middle layer Min temperature (°C)	4	8	6.143	1.245
SL_MaxT	Sub layer Max temperature (°C)	36	39	37.643	1.093
SL_MinT	Sub layer Min temperature (°C)	8	12	10	1.309
IR	Integrated radiation	1125	1351	1201.857	67.455

For the mentioned data, since pavement inspections are conducted once a year and rutting is a long-term-formed distress, the collection and processing methods for the data are as follows: The RDI was collected during the annual pavement inspection. Temperature data is aggregated on an annual basis, is derived from sensor records over the year, with maximum and minimum statistics calculated. While traffic data and maintenance funding data are obtained from the Foshan Ministry of Transportation (MOT) and further processed. Finally, all collected data is compiled and aggregated as input for the model.

**Fig 6:**
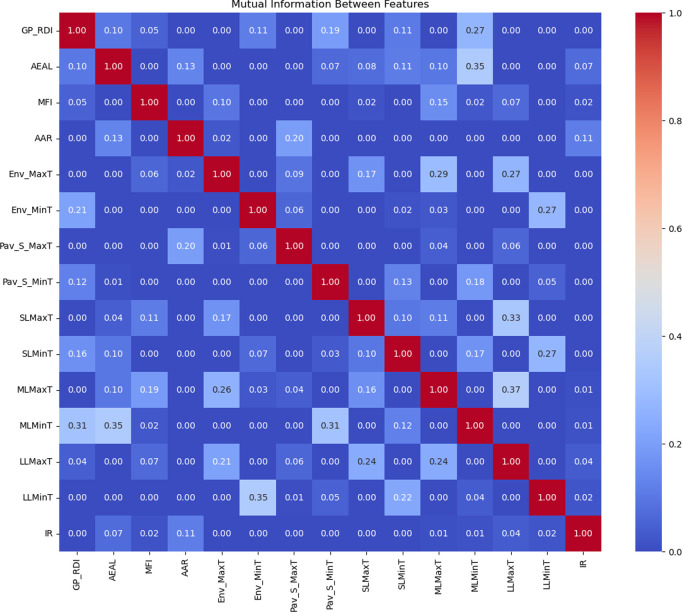
Mutual information analysis results of different features.

Meanwhile, to avoid interactions between variables, this study employs mutual information to examine the relevance among the variables. Mutual information is a method from information theory that measures the dependency between two variables, capturing both linear and nonlinear statistical associations. Compared to traditional correlation metrics (such as Pearson correlation coefficient), it has broader applications in data analysis and feature engineering. The results of the mutual information analysis for the 15 variables mentioned in Table 4 are shown in the Fig 6. The findings indicate that the mutual information values between the variables are generally low, with the majority of the variables being completely independent and a small subset exhibiting weak dependencies.

Furthermore, when applying machine learning techniques, to enhance the convergence speed, improve the training outcomes, and reduce computational resource consumption, the foregoing data is subjected to Z-score normalization. This process clusters the data within a specific range while compressing the extreme values into a smaller interval. When developing the pavement performance prediction model, all data will be normalized before being input into the model. This approach enhances model accuracy and reduces computational costs.

## Methods

### Gray forecast model

Gray prediction models are forecasting approaches that construct mathematical models and make predictions based on a limited and incomplete set of information. They have been extensively applied in the field of pavement performance prediction [18,25,38]. Among these models, the GM (1,1) model is one of the most commonly used method, characterized by its minimal requirement for modeling information, low dependence on historical data, and convenient computational process [39–41]. By integrating all known and unknown information into a single system for holistic analysis, it simplifies the impact of various complex factors within the system, leading to desirable computational outcomes. For RDI, whose deterioration is influenced by various stochastic factors such as traffic loads, climatic conditions, and material properties, resulting in significant data uncertainty. The GM(1,1) model effectively reduces randomness in the data by transforming raw observations into a monotonically increasing sequence through Accumulated Generating Operation, thereby extracting the overall trend of pavement performance degradation [18]. This makes GM(1,1) a suitable method for predicting pavement performance.

Initially, applying the GM (1,1) model to pavement performance prediction initially requires organizing the collected historical road surface data into a time series of Road Deterioration Index (RDI) values, given *x*^*(0)*^*(n)* as the RDI of year n, time series X^(0)^ can be denoted as Eq (1):


$$X^{\left(0\right)}=\left\{x^{\left(0\right)}\left(1\right),x^{\left(0\right)}\left(2\right),...,x^{\left(0\right)}\left(n\right)\right\}$$ (1)

Due to the stochastic and volatile nature of historical inspection data, the cumulative sum method should be employed to process the historical RDI data, shown as Eq (2), and resulting in a new time series *X*^*(1)*^, shown as Eq (3). This approach helps to smooth out the random fluctuations and provides a more stable basis for modeling and prediction.


$$x^{\left(1\right)}\left(k\right)=\sum_{i=1}^{k}x^{\left(0\right)}\left(1\right),k=1,2,...,n$$ (2)

$$X^{\left(1\right)}=\left\{x^{\left(1\right)}\left(1\right),x^{\left(1\right)}\left(2\right),...,x^{\left(1\right)}\left(n\right)\right\}$$ (3)

Consequently, using Eq (4) to calculate the neighboring mean z^(1)^(k) of time series X^(1)^ and obtained the neighboring mean sequence Z^(1)^, denoted as Eq (5).


$$z^{\left(1\right)}\left(k\right)=\left(x^{\left(1\right)}\left(k\right)+x^{\left(1\right)}\left(k-1\right)\right)/2,k=2,3,...,n$$ (4)

$$Z^{\left(1\right)}={\left\{z^{\left(1\right)}\left(2\right),z^{\left(1\right)}\left(3\right),...,z^{\left(1\right)}\left(n\right)\right\}}^{T}$$ (5)

Further, establishing GM (1,1) differential equation using time series X(0) and Z(1), denoted as Eq (6).


$$x^{\left(0\right)}\left(k\right)+az^{\left(1\right)}\left(k\right)=b$$ (6)

Solve this differential equation by applying ordinary least squares method, given A=[a, b]^T^, the solution equation is shown as Eq (7).


$$A={\left(N^{T}N\right)}^{-1}N^{T}Y$$ (7)

where $N=\left[\begin{array}{c} {}*20c-z^{\left(1\right)}\left(1\right)1\\ -z^{\left(1\right)}\left(2\right)1\\ \vdots \vdots \\ -z^{\left(1\right)}\left(n\right)1 \end{array}\right]$ and $Y=\left[\begin{array}{c} {}*20cx^{\left(0\right)}\left(2\right)\\ x^{\left(0\right)}\left(3\right)\\ \vdots \\ x^{\left(0\right)}\left(n\right) \end{array}\right]$.

We establish a one-stage albinism equation as Eq (8), based on which a time response function cumulative sequence value model can be obtained as Eq (9).


$$\frac{dX^{\left(1\right)}}{dt}+aX^{\left(1\right)}=b$$ (8)

$${\hat{x}}^{\left(1\right)}\left(k+1\right)=\left(x^{\left(0\right)}\left(1\right)-\frac{b}{a}\right)e^{-ak}+\frac{b}{a},k=1,2,...,n$$ (9)

Ultimately, the GM (1, 1) predictor is shown as Eq (10).


$${\hat{x}}^{\left(0\right)}\left(k+1\right)=\left({\hat{x}}^{\left(1\right)}\left(k+1\right)-{\hat{x}}^{\left(1\right)}\left(k\right)\right)$$ (10)

The previous section elaborated on the specific principles of the GM(1,1) model and its suitability for pavement performance prediction. However, this model is only applicable to monotonic change trends, and its linear assumption may fail to accurately capture the actual nonlinear degradation process. Consequently, relying solely on GM(1,1) for prediction has limited accuracy [25].

### BP neural network

Neural networks are currently the most common method used for regression prediction [42–44], where, the Backpropagation (BP) neural network is a multi-layer feedforward neural network trained according to the error backpropagation algorithm, is one of the most widely used neural network models [45]. Historically, this method has been extensively applied in the field of traffic forecasting, crash modelling and other research [43,46,47]. Compared to other common machine learning models such as RF, XGBoost, and LSTM, RF and XGBoost struggle to handle time-series-related data, while the LSTM model often requires a large amount of data for training, making them unsuitable for this study [48–50]. The advantage of this algorithm is its ability to represent the complex relationships between various influencing factors and the variables to be predicted, without the need to describe these relationships between inputs and outputs. Previous studies have indicated that the BP neural network has stronger adaptability and accuracy compared to traditional models [43,44].

**Fig 7:**
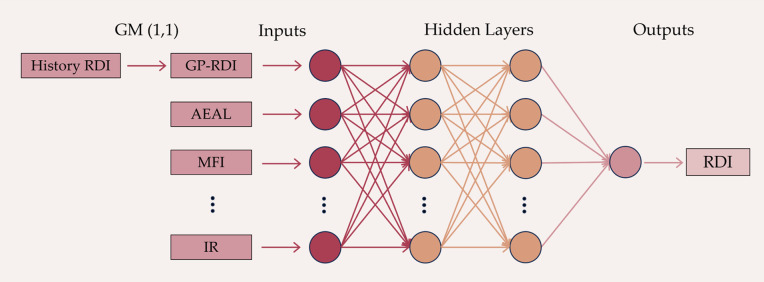
Overall schematic diagram of GM(1, 1) – BPNN predictor.

The signals in a BP neural network are propagated forward, while errors are propagated backward. During the computation process, the network’s weights and thresholds are continuously adjusted through backpropagation to minimize the sum of squared errors. The BP network consists of an input layer, hidden layers, and an output layer, as shown in Fig 7. Information enters the network at the input layer and passes through each intermediate layer’s calculations to reach the output layer, which is the forward propagation process. However, since the parameters are random, the result of the first calculation will have a significant error compared to the actual result. It is necessary to adjust the parameters based on the error to allow them to fit better until the error is minimized. This requires the model’s backpropagation, which primarily adjusts the internal parameters of the network by calculating the error between the output layer’s results and the expected values. In the process of road performance prediction, the relationship between road performance and its influencing factors is often nonlinear. Therefore, it is suitable to use the BP neural network (BPNN) to predict road performance based on environmental and traffic volume factors.

### Combined GM(1,1)-BPNN predictor

In the preceding sections of this paper, the Grey Prediction Model GM (1,1) and the Back Propagation Neural Network (BPNN) were introduced. Currently, the GM (1,1) model suffers from low accuracy in long-term forecasting and is unable to reflect the internal development laws of the system when subjected to random factors, leading to significant errors. Road performance is influenced by numerous factors, including climate and maintenance, hence relying solely on GM (1,1) for prediction may result in poor accuracy [25,51]. The BPNN possesses strong nonlinear, self-learning, and self-adaptive capabilities, enabling it to consider the impact of various influencing factors on the Rutting Depth Index (RDI) comprehensively [43]. Therefore, this paper integrates grey prediction with machine learning methods, employing a GM (1,1) – BPNN approach to forecast RDI. The overall structure of the predictor is illustrated in the Fig 7.

As shown in the Fig 7, the predictor first uses GM(1,1) to predict the trend of RDI changes based on historical RDI data. Further, the prediction results of GM(1,1) are used as independent variables along with other environmental variables shown in Table 4 and input into the BPNN model. Through the above steps, the future RDI values can be predicted by the GM(1, 1)-BPNN predictor.

### Model performance

To show the goodness of the proposed predictor, we compare the proposed GM (1,1)-BPNN predictor with GM (1,1), which have been introduced in the foregoing section and PPI model.

The PPI pavement serviceability deterioration equation is defined as follow Eq (11) [52].


PPI=PPI0{1−exp[−(\raise0.7ex\(\alpha \)/αy\nulldelimiterspace\lower0.7ex\(y\))β]} (11)

where PPI denotes the predicted RDI, PPI_0_ denotes the preliminary RDI value, y denotes the time period since the freeway open to traffic, α and β are mode coefficient, referenced to previous study, α = 13.2 and β = 1.409 [52].

The GM (1, 1)-BPNN proposed in this paper for predicting RDI tasks is a regression task. For such tasks, scholars have proposed a series of indicators to comprehensively evaluate the performance of predictors. The main ones are as follows:

Mean Absolute Error (MAE) [53]: This measures the average magnitude of the errors in a set of predictions, without considering their direction. It is calculated as the sum of the absolute differences between predicted and actual values divided by the number of observations, shown as Eq (12).


$$MAE=\frac{1}{n}\sum_{i=1}^{n}\left|f_{i}-y_{i}\right|$$ (12)

Mean Squared Error (MSE) [54]: This measures the average of the squares of the errors—that is, the average squared difference between the estimated values and the actual value. It is calculated as the sum of the squared differences between predicted and actual values divided by the number of observations, shown as Eq (13).


$$MSE=\frac{1}{n}\sum_{i=1}^{n}{\left(f_{i}-y_{i}\right)}^{2}$$ (13)

Root Mean Squared Error (RMSE) [53]: This is the square root of the mean of the squared errors. RMSE is a measure of the differences between values predicted by a model and the values actually observed. It is calculated as the square root of the MSE, shown as Eq (14).


$$RMSE=\sqrt{\frac{1}{n}\sum_{i=1}^{n}{\left(f_{i}-y_{i}\right)}^{2}}$$ (14)

Coefficient of determination (R²): This is a statistical measure that represents the proportion of the variance for a dependent variable that’s explained by an independent variable or variables in a regression model. The R-squared value ranges from 0 to 1, with 1 indicating that the regression predictions perfectly fit the data, shown as Eq (15).


$$R^{2}=1-\frac{\sum {\mathrm{\backslash nolimits}}_{i=1}^{n}{\left(f_{i}-y_{i}\right)}^{2}}{{\sum {\mathrm{\backslash nolimits}}_{i=1}^{n}\left(y_{i}-\bar{y}\right)}^{2}}$$ (15)

where, *n* denotes the number of sample, *f*_*i*_ denotes the predicted value of RDI, *y*_*i*_ denotes the measured value of RDI, ‾*y* denotes the mean value of measured RDI.

Considering the data characteristic of this study, this study utilizes MSE to describe the model performance.

### SHAP techniques

SHAP (SHapley Additive exPlanations) is a method for explaining the prediction results of machine learning models [55–57]. It is based on the concept of Shapley values in game theory, assigning importance values to each feature of the model to explain the model’s prediction process, proposed in 2017 [58]. Compared to traditional mathematical models, machine learning models were considered a black box, where scholars could only input variables to obtain outputs without understanding the relationship between the inputs and the outputs, including the quantitative relationship and the degree of influence. SHAP introduces Shapley values from cooperative game theory and calculates the contribution of each feature to the model’s prediction results based on these values. To calculate the SHAP values of features, the SHAP algorithm considers all possible combinations of features and calculates the prediction differences between the current set of features and the set without a particular feature. Then, it averages the differences from all possible feature combinations to derive the SHAP value for that feature. This method can explain the prediction of a single sample by showing the contribution of each feature to the explained variable, and it can also provide an understanding of the overall impact of the variables on the model by analyzing the distribution of SHAP values for all features.

To calculate the SHAP of feature i, two models should be trained. Given set *F* consists of all n features, and subset *S* consists of n-1 (except for feature i) features. The first model is trained with all n features in set F, marked as *f*_S∪{i}_(*x*_S∪{i}_) while the second one is trained with n-1 features in set S, marked as *f*_S_(*x*_S_). By calculating the different between the outputs of two models, the SHAP value of feature i can be obtained as Eq (16).


$$S_{i}=\sum_{S\subseteq F\backslash \{i\}}\frac{|S|!\left(|F|-|S|-1\right)!}{|F|!}\left(f_{S\cup \{i\}}\left(x_{S\cup \{i\}}\right)-f_{S}\left(x_{S}\right)\right)$$ (16)

SHAP interpretability analysis plays a pivotal role in providing comprehensive local and global interpretability information. A positive SHAP value signifies a favorable impact of the variable on the output while a negative SHAP value denotes an adverse effect of the variable.

## Results

### Model performance

Before building the model, the collected data used for training was first checked for missing or extreme values. After employing boxplot tests and data processing, it was confirmed that the dataset used in this study contains no missing or extreme values, making it suitable for training the GM (1, 1)-BPNN combined predictor.

Based on previous research, this paper initially divided the whole dataset into two subsets. The data obtained between 2015–2019 were treated as training set, while data obtained in 2020–2021 were treated as validation set. According to the description in the Methodology section, we first used the GM (1, 1) model to forecast the pavement RDI, obtaining the GM_RDI as the input data for the BP neural network. For the hyperparameter of the proposed model, there are a total of 15 factors related to the performance of asphalt pavements, hence the number of input layer units is 15. As shown in Table 4, there are a total of 15 factors influencing asphalt pavement performance, thus the input layer size is set to 15 units. For the number of hidden layers and learning rate, an exhaustive search method was employed to select the best-performing combination. After experimentation, the final configuration adopts 1 hidden layer with 6 units.

For the activation function, previous research indicates that the ReLu function performs better in nonlinear problems [43], therefore, it is chosen as the activation function. The model contains a total of 102 weight and threshold parameters. The training process is configured with 100 epochs and the EarlyStopping callback (with patience = 5 and min_delta = 0.01). The learning rate is set to 0.0025, an optimal value determined through repeated testing. Training is terminated if no improvement in the monitored metric is observed. In this study, the model stopped training at the 15th epoch.

At the end of training, the model achieved the following performance metrics on the training set. The MAE, MSE, RMSE and R^2^ are 0.146, 0.415, 0.172 and 0.91, respectively.

Based on the comparative evaluation of the three models (GM(1,1)-BPNN hybrid model, GM(1,1), and PPI model) on the test set, their performance metrics are summarized in Table 5.

**Table 5:** Model performance on validation set.

Model	MAE	MSE	RMSE	R^2^
PPI	13.045	173.878	13.186	0.12
GM (1, 1)	0.280	0.083	0.289	0.77
**GM(1, 1)-BPNN**	0.068*	0.004*	0.068*	0.79*

As shown in Table 5, the proposed hybrid model achieves an MAE of 0.068, MSE of 0.004, RMSE of 0.068, and R² of 0.79, outperforming the baseline GM(1,1) model and the PPI model. Notably, the PPI model demonstrates the weakest performance among the three. Overall, both the GM(1,1) model and the hybrid GM(1,1)-BPNN model exhibit smaller errors and strong nonlinear fitting capabilities when compared to historical RDI measurements. However, the hybrid model demonstrates superior accuracy, enabling more precise tracking of actual pavement deterioration trends.

### SHAP analysis

**Fig 8:**
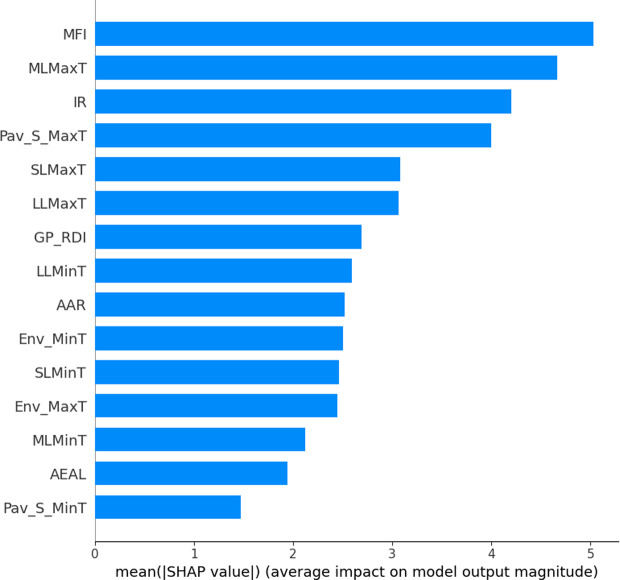
Feature significant result.

**Fig 9:**
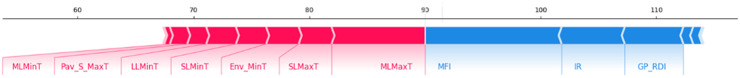
SHAP force plot of RDI of 2016.

In this section, we further use SHAP technique to interpret the output of the preferred model, GM(1,1)-BPNN. Based on the introduction in Section 4.5, the research team calculates the SHAP value of each feature using SHAP module in Python and results are displayed in Fig 8 and 9. For the features name of the abbreviations, readers can refer to Table 4 for more information.

Fig 8 illustrates the ranking of the importance of various features. The ranking is determined based on the SHAP values of different features as calculated using Eq. (specific equation reference). As shown in the Fig 8, Maintenance Fund, Middle Layer Maximum Temperature, Integrated Radiation, and Pavement Surface Maximum Temperature have the most significant impact on RDI. Additionally, Upper Layer Maximum Temperature, Sub-Layer Maximum Temperature, Rutting Depth Index predicted by GM(1,1), and Sub-Layer Minimum Temperature also play a critical role in influencing RDI.

To further explore the specific impacts of various factors on the RDI, the SHAP force plot for 2016 RDI values was generated using Python, as shown in Fig 9. The plot reveals that factors such as Maintenance Fund, Integrated Radiation, and Rutting Depth Index predicted by GM(1,1) are associated with higher RDI values. Conversely, factors like Middle Layer Max Temperature, Upper Layer Max Temperature, Environmental Min Temperature, Upper Layer Min Temperature, Sub-layer Min Temperature, Pavement Surface Max Temperature, and Middle Layer Min Temperature are linked to lower RDI values, indicating a negative influence on pavement serviceability.

## Discussions

### Model performance

According to the model fitting results, the proposed model demonstrates superior performance across all metrics. This is primarily attributed to its integration of the strengths of both the GM(1,1) grey prediction model and the BP neural network. As mentioned in the Methods section, the GM(1,1) model is well-suited for predicting small-sample, highly uncertain data, as its core approach involves modeling data through accumulated sequence generation, allowing it to effectively capture the overall trend of the data [18,25]. On the other hand, the BP neural network possesses powerful nonlinear mapping capabilities, enabling it to learn the intrinsic patterns of the data and compensate for the GM(1,1) model’s limitations in detailed fitting [43]. This combined approach not only accounts for the overall data trend but also refines local fluctuations through the neural network, resulting in more accurate and stable predictions.

As for the standalone GM(1,1) and PPI models. The PPI (Performance Prediction Index) model is primarily used for predicting pavement performance based on road age and regional factors. Its core assumption is that pavement performance decays exponentially over time, with regional factors playing a significant role in this degradation. However, the model’s limitations lie in its exclusive focus on road age and regional factors while neglecting other dynamic variables that may impact pavement performance, such as traffic load, weather conditions, and material deterioration. As a result, its accuracy diminishes significantly over longer prediction periods, leading to substantial deviations between predicted and actual values, particularly in scenarios with pronounced short-term fluctuations. Meanwhile, the GM(1,1) model, as a classic grey forecasting method, is suitable for small-sample, incomplete-information data prediction, excelling at capturing overall data trends. However, its performance is limited when dealing with complex nonlinear data, as it struggles to reflect detailed local fluctuations—such as annual temperature variations or specific road maintenance investments by management agencies. While its prediction accuracy is superior to that of the PPI model, it still falls short of the combined model’s performance.

### SHAP analysis

These findings align with the practical engineering experience of field professionals as well as conclusions from previous studies. For example, Maintenance Fund and Rutting Depth Index predicted by GM(1,1) are associated with higher RDI values, while Middle Layer Max Temperature, Upper Layer Max Temperature, Environmental Min Temperature, Upper Layer Min Temperature, Sub-layer Min Temperature, Pavement Surface Max Temperature, and Middle Layer Min Temperature are linked to lower RDI values, indicating a negative influence on pavement serviceability. The above results are consistent with the empirical knowledge of field engineers as well as the conclusions drawn in previous research [27–29]. Adequate maintenance funding enables highway authorities to utilize higher-quality materials, more advanced equipment, and specialized techniques for pavement maintenance and rehabilitation. Additionally, excessive high and low temperatures negatively impact different pavement layers. Elevated temperatures may soften asphalt pavements, accelerating rutting, while low temperatures contribute to other forms of distress (e.g., cracking), which may further exacerbate rutting when temperatures rise again.

Notably, the positive correlation between radiation intensity and RDI contrasts with earlier research conclusions [59–61], we believe this phenomenon primarily stems from the fact that the road studied in this research is located in Foshan City, Guangdong Province, China—a region characterized by a typical subtropical monsoon climate. Due to the area’s consistently high temperatures, the measured annual average solar radiation levels remain elevated. This results in the RDI (rut depth index) being more susceptible to the influence of other factors in the model analysis, potentially leading to counterintuitive research conclusions. However, this does not contradict the established understanding that radiation levels can negatively impact RDI. To comprehensively investigate the influence of radiation on RDI, future studies should incorporate data from roads in diverse geographic regions, enabling a more objective assessment of radiation’s effects.

Moreover, to the best of the authors’ knowledge, previous studies have rarely analyzed the influence of temperature variations across different pavement layers on road service performance. This study reveals that, in addition to environmental and pavement surface temperatures, the internal temperatures of asphalt layers also exhibit a significant correlation with pavement serviceability. Therefore, future research could further investigate the impact of internal layer conditions on pavement performance, aiming to provide a more comprehensive understanding of the deterioration mechanisms and influencing factors in asphalt pavements.

### Engineering application

This section evaluates the engineering applicability of the proposed model. In terms of prediction accuracy and stability, the GM(1,1) – BPNN hybrid model demonstrates superior performance by effectively integrating multiple influencing factors and better handling nonlinear data characteristics. Consequently, its predictions are more precise and reliable. In contrast, the PPI model and the standalone GM(1,1) model exhibit poorer long-term prediction performance due to their inherent limitations. This is particularly evident when dealing with complex and dynamic pavement performance data, where the hybrid model’s advantages become more pronounced, offering more robust technical support for scientific pavement management and maintenance.

However, the proposed model requires a set of relatively difficult-to-measure input data, such as temperature variations across pavement layers, which, as in this study, necessitates sensor deployment for collection. As such, this type of data is relatively hard to obtain in practical engineering scenarios. Therefore, management authorities should select an appropriate model based on actual maintenance needs. When conditions permit the collection of diverse data, the GM(1,1)-BPNN hybrid prediction model proposed in this study is recommended. On the other hand, when data acquisition proves challenging, the standalone GM(1,1) model can still achieve reasonable accuracy and meet basic functional requirements.

By comparing the replicability, comprehensiveness, forecasting period, and accuracy of the three prediction models, Table 6 summarizes the models. In the future, the appropriate model can be selected based on the actual situation of the data.

**Table 6:** Model summary.

Model	Replicability	Comprehensiveness	Forecasting period	Accuracy
GM (1,1)-BPNN	Fair	Excellent	Excellent	Excellent
GM (1,1)	Good	Fair	Good	Good
PPI	Excellent	Good	Fair	Fair

To provide scientific pavement maintenance basis for freeway pavement administration, we selected 3 years as pavement maintenance period to predict future pavement RDI condition. Initially, the GM(1,1) predicted RDI was calculated, then this value and other features were input into the trained combined predictor. Subsequently, the predicted value of RDI in the latter three years can be obtained. After calculation, the predicted RDI value of Freeway A in 2022, 2023 and 2024 are 91.96, 90.59 and 88.08 respectively.

The results indicate that the Rut Depth Index (RDI) of the studied freeway exhibits a declining trend year by year. In practical operations, management authorities can leverage historical environmental data and projected pavement maintenance budgets to forecast RDI trends over future years. Based on these projections, prioritized maintenance sections can be identified by evaluating both current RDI values and their rate of deterioration.

## Conclusions

This study proposed a combined pavement serviceability prediction model combining GM(1,1) and BP neural networks. Using a highway in Guangdong Province as a case study, sensors were installed along the road. Based on seven years of data from 2015 to 2021, the pavement RDI values of this highway were predicted and compared with the standalone GM(1,1) grey prediction model and the PPI pavement deterioration model. Furthermore, the SHAP analysis method was employed to interpret the model and analyze the influencing factors, leading to the following main conclusions:

(1) By installing sensors, data on environmental factors, road surface, and layers (upper, middle, and lower) were collected, including annual maximum and minimum temperatures, annual axle load counts, maintenance funding, and cumulative annual radiation. The results indicate that all these factors significantly influence the pavement RDI values.
(2) The proposed combined model has a higher prediction performance. Validated by validation set, the MAE, MSE, RMSE as well as R^2^ were 0.068, 0.004, 0.068, 0.79, respectively, surpassing the baseline model PPI and GM(1,1);
(3) Compared with the standalone GM(1,1) model and the PPI model, the proposed model outperformed in terms of considering more factors, prediction duration, and accuracy, though it was less operationally convenient than the two baselines. The predicted RDI of the research freeway in 2022, 2023 and 2024 are 91.96, 90.59 and 88.08, respectively. The pavement administration can formulate scientific pavement maintenance strategy based on the predicted results.
(4) SHAP analysis revealed that maintenance funding, middle-layer maximum temperature, annual radiation, surface maximum temperature, upper-layer maximum temperature, and lower-layer maximum temperature were the most influential factors on RDI values.

This study provides a scientific reference for highway pavement management units to develop maintenance plans, contributing to precise planning and cost savings. In the future, sensors could be deployed across multiple regions and road segments to gather more data and further optimize the model.
